# Molecularly Imprinted Nanofiber Film for Sensitive Sensing 2,4,6-Tribromophenol

**DOI:** 10.3390/polym8060222

**Published:** 2016-06-22

**Authors:** Limei Huang, Meishan Li, Dan Wu, Xiuling Ma, Zhenyue Wu, Shengchang Xiang, Sheng Chen

**Affiliations:** 1 College of Chemistry and Chemical Engineering, Fujian Provincial Key Laboratory of Polymer Materials, Fujian Normal University, Fuzhou 350007, China; lmhuanghx@126.com (L.H.); limeishan2015@163.com (M.L.); wudan2521275@163.com (D.W.); 18950421622@163.com (Z.W.); scxiang@fjnu.edu.cn (S.X.); 2 School of Ocean Science and Biochemistry Engineering, Fuqing Branch of Fujian Normal University, Fuqing 350300, China

**Keywords:** molecular imprinting, electrospinning, 2,4,6-tribromophenol, electrochemistry sensor

## Abstract

The determination of brominated flame retardants is of great importance, but remains a challenge. Particularly, universal and facile approaches are limited. Here we report a new general approach, combining molecular imprinting and electrospinning, for the efficient and facile imprinting sensor of 2,4,6-tribromophenol (TBP), which was used as a “novel” brominated flame retardant. With TBP as the template molecular, β-cyclodextrin (β-CD) as the functional monomer, and poly-vinylbutyral (PVB) as the electro-spinning matrix, the nanofiber film was deposited on the glassy carbon electrode (GCE) via electrospinning technique directly. The β-CD-PVB/GCE sensor system exhibited excellent TBP sensing performances, such as a low detection limit (6.29 × 10^−10^ mol·L^−1^) at room temperature, selective recognition to TBP/phenol/4-methyl-phenol, and good regeneration performance. The approach of fabricating a molecular imprinting nanofiber sensor may shed new light in the detection of other phenolic pollutants.

## 1. Introduction

Brominated flame retardants (BFRs) have been routinely added to a variety of consumer and industrial products for several decades [1,2]. Some of the BFRs are stable, bioaccumulative, capable of long distance transport in the environment, and potentially harmful to ecosystems and human health [3,4]. This has paved the way for the use of “novel” BFRs (NBFRs) as replacements for the banned formulations. Recently, a number of these NBFRs are of particular concern as they are being found in the Arctic, indicating long-range atmospheric transport (LRAT) [5,6].

2,4,6-Tribromophenol (TBP) has been used as a NBFR or a synthetic intermediate of most of the important BFRs [7]. It is ubiquitously found in aquatic environments and biota [8,9,10]. What is more, there is some information on TBP toxicity and its effects on humans and the environment. For example, TBP caused an induction of aromatase activity in the human adrenocortical (H295R) cell line [11]; chronic exposures to environmental levels of tribromophenol impair zebrafish reproduction [12]. Therefore, the detection of TBP has attracted considerable attention. Recently, methods to detect TBP in the environment were almost established by gas chromatography (GC) coupled with mass spectrometry (MS) or electron capture (EDC) with good sensitivity [13,14,15]. These protocols need relatively expensive equipment, and it is not conducive to the emergency and simple monitoring of environmental pollutants. Hence, the important and challenging task is to develop a simple, sensitive, and selective method for on-site determination of TBP.

Sensor technology has attracted considerable attention due to its good selectivity, low cost, and simple operation. In order to improve the sensitivity of sensors, the current trend is to produce nanoscale sensors [16]. Among various methods to build nanoscale structures, electrospinning is rapidly emerging as a facile and versatile approach to produce multifunction nanofibers with controllable morphology [17,18,19], with the reported nanosensor exhibiting satisfying sensing properties [20,21].

In recent years, molecular imprinting technology (MIT) has been a fascinating area of scientific research because it has been proven to be the most efficient way of making synthetic materials bearing selective molecular recognition sites [22]. Molecular imprinting polymers (MIPs) are prepared by a process that involves co-polymerization of functional monomers and cross-linkers around template molecules. The molecules are removed from the polymer, rendering complementary binding sites capable of subsequent template molecule recognition [23]. Based on MIPs with affinity and specificity, they possess many promising characteristics, such as desired selectivity and easy preparation. Therefore, MIPs have been an increasing concern for applications [24,25,26,27,28], particularly for the use as the recognition element in the sensor [29,30,31,32]. Up to now, the methods of fabricating the imprinted sensor were mainly developed by (a) electropolymerization in electric conductive material, such as a glassy carbon electrode surface; (b) *in situ* photopolymerization by the monomer mixture spin coating on the surface of the converter; and (c) preparing nanometer- or micron-sized MIP particles, then mixing the particles and inert soluble polymer, then fixing the mixture on the surface of the converter. If the sensor is fabricated via incorporating molecular imprinting with electrospinning, it might provide a perfect effect on the selectivity and sensitivity of the sensors.

In this paper, a facile strategy to incorporate MIT with electrospinning and electrochemical techniques is proposed to sense TBP, which is trace in water. With poly-vinylbutyral (PVB) as the electro-spinning matrix, β-cyclodextrin (β-CD) as the functional monomer, and TBP as the template molecule, the β-CD-PVB nanofiber film was deposited on the glassy carbon electrode (GCE) via electrospinning technique directly, which could retain the intrinsic high specific surface area of β-CD-PVB nanofibers and significantly simplify the TBP detection process. β-CD is chosen as the functional monomer for imprinted nanofibers because of its special properties and ease of electrospinning with various polymer materials to form nanofibers with good chemical and mechanical stability [33,34]. The β-CD-PVB/GCE sensor system exhibited excellent TBP sensing performance, such as good selective recognition to TBP/phenol/4-methyl-phenol and low detection limits of 6.29 × 10^−10^ mol·L^−1^ (0.21 µg·L^−1^). Our research might provide new insight to construct the molecularly-imprinting nanofiber sensor to sensitively sense other phenolic pollutants.

## 2. Experimental

### 2.1. Instruments and Reagents

Morphology of the nanofibers was characterized by SEM (JSM-7500F, JEOL, Tokyo, Japan). The thermal stability of fibers was characterized by thermal gravimetric-differential thermal analysis (TG/SDTA851e, Mettler-Toledo, Zurich, Sweden). A CHI660E electrochemical workstation (CHI Instrument, Shanghai Chenhua Apparatus Company, Shanghai, China) was used for electrochemical measurements.

PVB (aerial grade, butyral content: 66%–75%) and β-CD were obtained from Guoyao Chemicals Co., Ltd (Shanghai, China). TBP and hexamethylene diisocyanate (HMDI) were obtained from Aladdin Chemistry Co., Ltd (Shanghai, China). All other chemicals were analytical reagent grade or better.

### 2.2. Fabrication of the Imprinted Nanofiber Sensor (INS)

**Figure 1:**
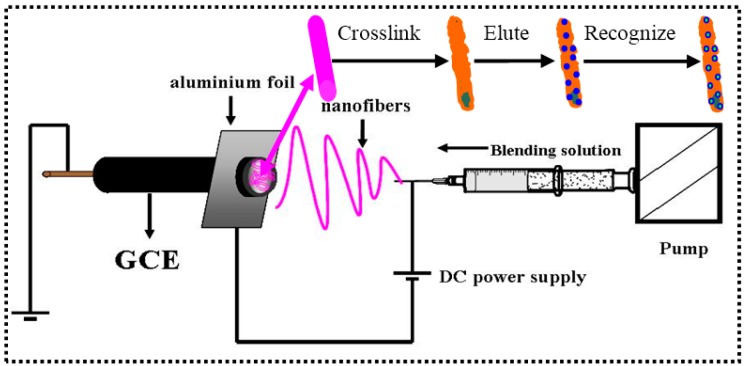
Schematic illustration of the fabrication of INS.

As shown in Figure 1, the INS was fabricated by the following description.

#### 2.2.1. Preparation of the Composite Nanofibers

Similar to [33], the PVB/β-CD/TBP solution was obtained by dissolving PVB, β-CD, and TBP in *N*,*N*-dimethylformamide (DMF). The PVB concentration was of 0.08 g·mL^−1^, a β-CD concentration of 0.04 g·mL^−1^, and the TBP:β-CD molar ratio was 1:5. The resulting PVB/β-CD/TBP mixture was stirred at room temperature to obtain a homogenous mixture with viscosity suitable for electrospinning.

Electrospinning was carried out at room temperature. The solutions were placed in a 10 mL syringe fitted with a metallic needle of 0.4 mm inner diameter. The syringe was fixed horizontally, and a stainless steel electrode was connected to a high-voltage power supply (Tianjin Technical Corp, Tianjin, China). The applied voltage between the tip and collector was 18 kV, with a tip-to-collector distance of 10 cm. The flow rate of the solution was controlled by a syringe pump at a constant rate of 0.5 mL·h^−1^. The polished GCE was used as the collector. Prior to modification, GCE was polished with emery paper and chamois leather containing 0.3 and 0.05 µm Al_2_O_3_ slurry, respectively, and then thoroughly rinsed ultrasonically with HNO_3_, ethanol, and doubly-distilled water for 3 min, in turn. Then, the electrode was cycled between −0.3 and 1.5 V in 0.5 mol·L^−1^ H_2_SO_4_ at a 100 mV·s^−1^ scan rate so that a reproducible voltammogram was obtained.

#### 2.2.2. Cross-linking and Elution of Nanofibers

The PVB/β-CD/TBP nanofiber was immersed into HMDI at room temperature for 24 h. Then, the template molecule TBP was removed from the resultant nanofiber with ethanol by shaking.

As a comparison, the non-imprinted nanofiber sensor (NINS) was also prepared by the same method as the preparation of INS, just without the addition of the template molecule TBP.

### 2.3. Electrochemical Measurements 

A conventional three-electrode system was employed with a modified glassy carbon electrode (GCE, 3.0 mm in diameter) as the working electrode, a platinum electrode as the counter electrode, and an Ag/AgCl electrode with saturated KCl as the reference electrode [35,36]. All potentials reported in this article were referenced to the Ag/AgCl electrode. Hexacyanoferrate (K_3_[Fe(CN)_6_]) is chosen as the redox probe of the INS/NINS sensors in solutions because TBP is electroinactive over the studied potential range. All measurements were carried out at room temperature.

The amperometric *i*-*t* curve (AC) (potential: 0 V) was utilized to evaluate the response to different substances. The electrochemical performance of INS/NINS sensors was studied by cyclic voltammetry (CV) and electrochemical impedance spectroscopy (EIS). Differential pulse voltammetry (DPV), which is relatively sensitive compared to the conventional CV method, was employed for the determination of the imprinting effect. CV, DPV, EIS, and AC experiments were performed in the 10 mL 5.0 mmol·L^−1^ K_3_[Fe(CN)_6_] with the addition of 0.1 mol·L^−1^ KCL as a support electrolyte.

## 3. Results and Discussion

### 3.1. Preparation of the INS

**Figure 2:**
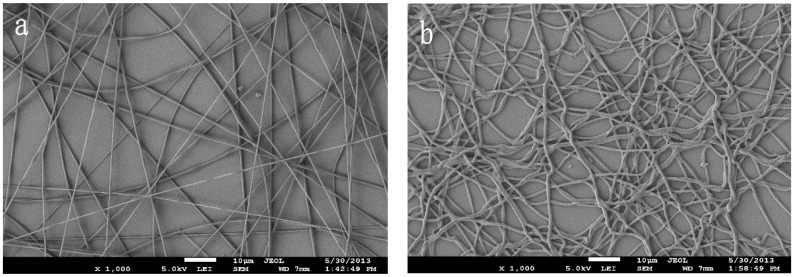
SEM morphology of nanofibers ((**a**) before cross-linking; and (**b**) after cross-linking).

The INS was fabricated by the combining of MIT and electrospinning, pioneered by Ye *et al.* [37], and recognized as an efficient technique allowing the creation of imprinting nanofibers for the affinity separation materials [38,39,40]. Similar to [34], the nanofibers were cross-linked by using HMDI. On the basis of SEM microphotographs of the nanofibers, a comparison can be made for nanofibers in terms of the morphological differences. It is observed that PVB/β-CD/TBP nanofibers have uniform diameters and are very straight in Figure 2a. However, it is interesting to find that the nanofibers cross-linked by HMDI are curly and adhered together at some sites (Figure 2b). This may be attributed to the flexible hexamethylene chain in HMDI.

**Figure 3:**
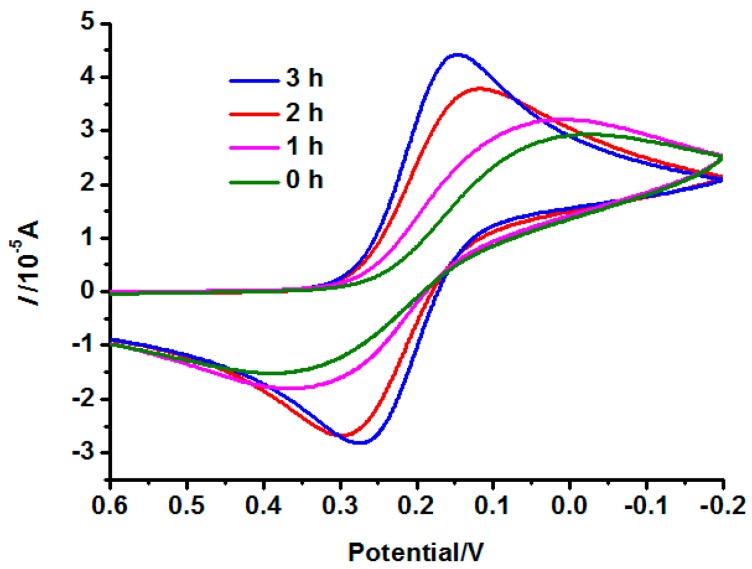
Elution of the molecular imprinted electrode by shaking.

CV was used to track the elution process. As shown in Figure 3, due to the dense films coated on the surface of the electrode before elution, almost no electrochemical reaction occurred, while, with the increase of the elution time, the peak current increases. Removing the imprinting molecule is an important factor when preparing MIPs. The imprinted cavity matched with the imprinting molecule will be formed after eluting the temple molecule. After elution, the electrochemical reaction occurs by a [Fe(CN)_6_]^3−^ ion through the imprinted cavities into the imprinted electrode surface. The differences before and after elution were shown by SEM and TG in a later section.

**Figure 4:**
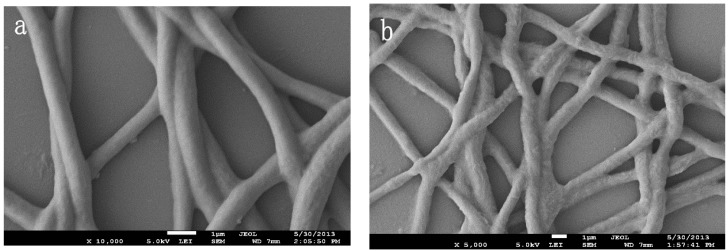
SEM morphology of molecular imprinted nanofibers after crosslinking ((**a**) before elution; and (**b**) after elution).

Compared with nanofibers before elution (Figure 4a), the nanofibers after elution (Figure 4b) are still curly, but more rough and loose. These changes are due to the removal of the template molecule. After eluting imprinting molecules, cavities left in the imprinted nanofibers may perfectly match with imprinting molecules.

**Figure 5:**
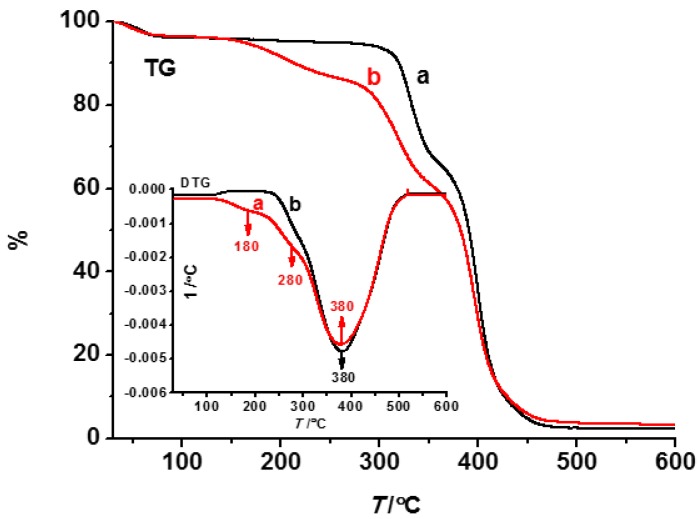
TG-DTG curves of nanofibers ((**a**) before elution (red line); and (**b**) after elution (black line)).

Figure 5 shows TG-DTG thermal stability curves of the composite fibers obtained at 18 kV. The weight decreased substantially in the low temperature region, which was attributed to the dehydration. In curve a, there were three further weight loss steps. A gentle peak at 180 °C in the DTG curve was attributed to the decomposition of TBP. The peak at 280 °C was assigned to the decomposition of β-CD. There was a weight loss of ~50% at 380 °C, which was assigned to the decomposition of PVB. After elution (curve b), no thermal decomposition peak of TBP emerged, except PVB and β-CD.

### 3.2. Electrochemical Properties of the INS

**Figure 6:**
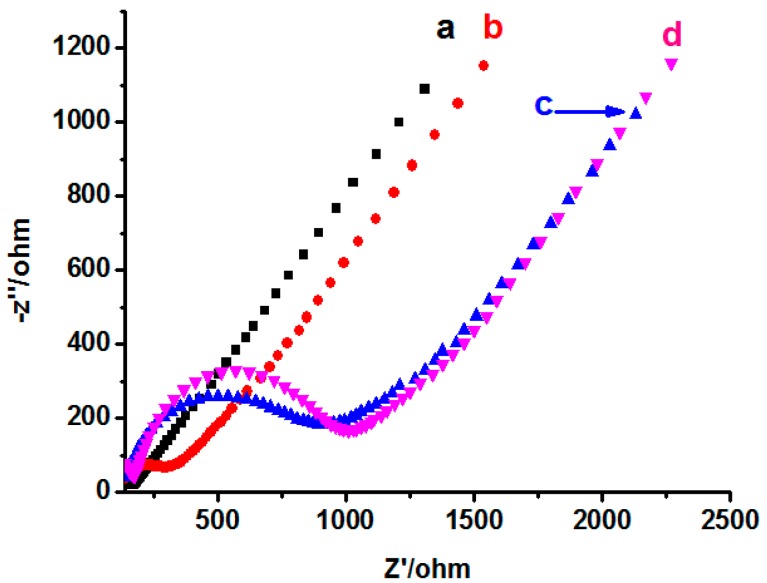
EIS of different electrodes ((**a**) bared electrode; (**b**) INS after elution; (**c**) NINS after elution; and (**d**) INS before elution).

EIS is an efficient tool for studying the films regarding the interfacial electron transfer kinetics. To characterize the surface property of the sensors, EIS (Init *E* = 0 V, High Freq = 1 × 10^4^ Hz, Low Freq = 1 Hz, Amplitude = 0.005 V) was used to investigate the change of resistance of different electrodes. As shown in Figure 6, the impedance value of bare GCE is minimal (curve a), and the diameter of semicircles of NINS electrode is larger than that of the INS (after elution), equal to the curve d (INS before elution). The result manifests that the imprinted one had a lower electron transport barrier presumably due to the existence of porous binding sites after the template removal. The decrease in interfacial impedance is desirable because it can decrease both the interfacial resistance drop of the electrode current and overpotential, leading to the enhancement of sensorial sensitivity [41].

**Figure 7:**
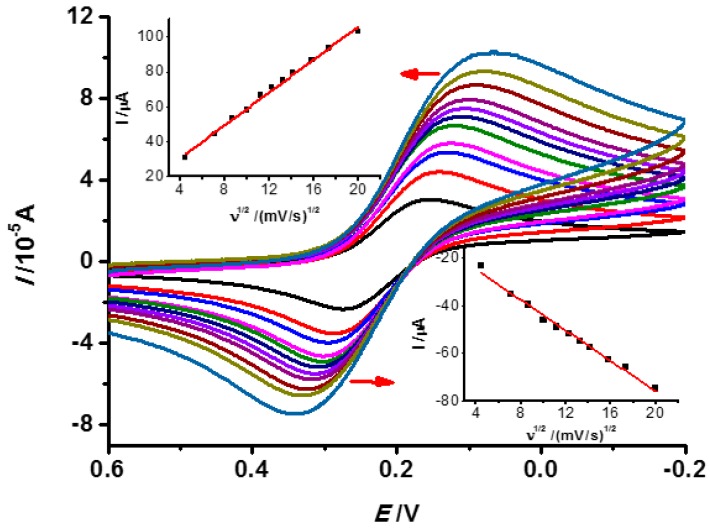
CV of the INS at various scan speeds of 20, 50, 75, 100, 125, 150, 175, 200, 250, 300, and 400 mV/s (from inner to outer), inset shows the calibration plot of *I*~*v*^1/2^.

Useful information involving electrochemical reactions can usually be obtained from the relationship between the peak current and scan rate. Therefore, the influence of scan rate on the INS was investigated by CV. As shown in Figure 7, the peak current increased with the scan rate over the range of 20~400 mV·s^−1^ and a good linear relationship between the anodic peak current and the square root of scan rates (*v*^1/2^) was obtained. The regression equation was *I*_pa_ = −3.18 *v*^1/2^ − 12.0 with a correlation coefficient of 0.9886. Moreover, the cathodic peak current was strongly dependent on the scan rate and the regression equation was *I*_pc_ = 4.71 *v*^1/2^ + 11.89 with a correlation coefficient of 0.9936. This indicated the reaction of imprinted sensor is a typical diffusion controlling process.

### 3.3. Effect of Imprinting of INS

**Figure 8:**
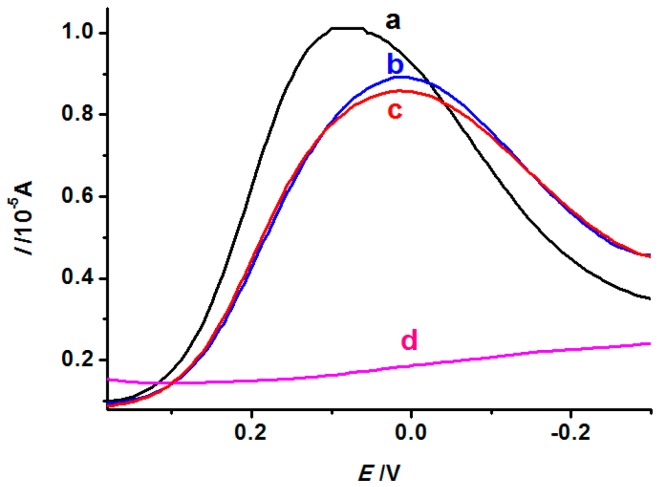
DPV responses of the INS to different concentration of TBP (**a**: 1.0 × 10^−7^; **b**: 1.0 × 10^−5^; **c**: 1.0 × 10^−3^ mol·L^−1^ and NINS to 1.0 × 10^−5^ mol·L^−1^ TBP (**d**)).

In this study, DPV was performed after the INS was immersed in solutions containing TBP of different concentrations (1.0 × 10^−7^, 1.0 × 10^−5^, and 1.0 × 10^−3^ mol·L^−1^) and the background solution (5 mmol·L^−1^ K_3_[Fe(CN)_6_]). When the INS was immersed in the solution containing TBP, the cavities in the film were partially occupied by TBP, which led to the decrease of current signal produced by [Fe(CN)_6_]^3−^. As shown in Figure 8, the higher the concentration of TBP (from curve a to curve c), the lower the current would be, which suggests that more and more binding sites in the film are occupied by TBP molecules. Additionally, the experiment on the NINS immersed in 1.0 × 10^−5^ mol·L^−1^ TBP solutions was also carried out (curve d). For NINS, due to no imprinting molecule, even after elution, its surface is still a layer of compact electrospinning film, which lacks cavity-containing binding sites. [Fe(CN)_6_]^3−^ is difficult to infiltrate into the electrode surface through the compact film, and curve d is relatively flat.

### 3.4. The Detection Limit of the INS

**Figure 9:**
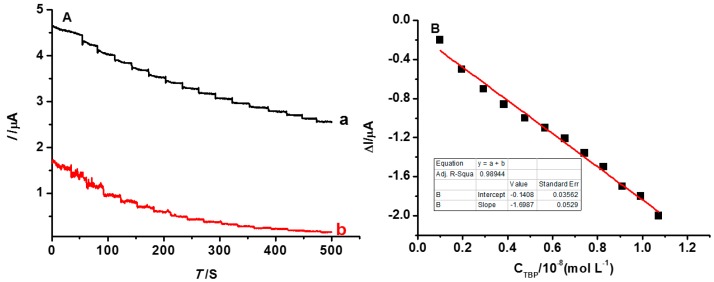
(**A**) is AC of the sensors (a: INS, b: NINS); and (**B**) shows the linear relation of ∆*I*~C_TBC_.

The detection limit of the INS was evaluated by the measurements of AC, which were performed in the 5 mL 5 mmol·L^−1^ K_3_[Fe(CN)_6_] solution, with 0.05 mL 1.0 × 10^−7^ mol·L^−1^ TBP added at the same time. As is shown in Figure 9, the current value of NINS is always lower than INS because the [Fe(CN)_6_]^3−^ ion is blocked from diffusing to the electrode by the dense surface structure of NINS. The current of K_3_[Fe(CN)_6_] gradually decreased with increasing TBP concentrations (Figure 9, curve a). It appeared as a platform at every concentration stage and stability in ~30 s, which indicated that the INS sensor has a short response time. When the imprinted electrode was immersed into the mixture solution, template molecules could be absorbed because of the imprinting spatial similarity. Therefore, it causes the variation of the K_3_[Fe(CN)_6_] peak current response. The relative change of the peak current is linearly proportional to the TBP concentration in the range of 9.9 × 10^−10^ mol·L^−1^ to 1.071 × 10^−8^ mol·L^−1^ (shown in Figure 9B), with a correlation coefficient of 0.98944. The limit of detection (LOD, LOD = 3 S/m, where S is standard deviation of current value, and m is sensitivity, which is the slope of the linear equation.) was 6.29 × 10^−10^ mol·L^−1^ (0.21 µg·L^−1^). The LOD of the imprinted nanofiber-coated electrode is *ca.* 23 times smaller than that of the imprinted core-shell nanoparticles one (4.98 µg·L^−^^1^) [35]. What is more, it is outperformed more than that of GC (0.5 µg·L^−1^) [15]. According to the hygiene standard, the content of phenol in drinking water is for 2 µg·L^−1^ or less, and the INS we proposed is desirable for the detection of trace amounts TBP in environmental samples.

### 3.5. The Selectivity Performance of the INS

**Figure 10:**
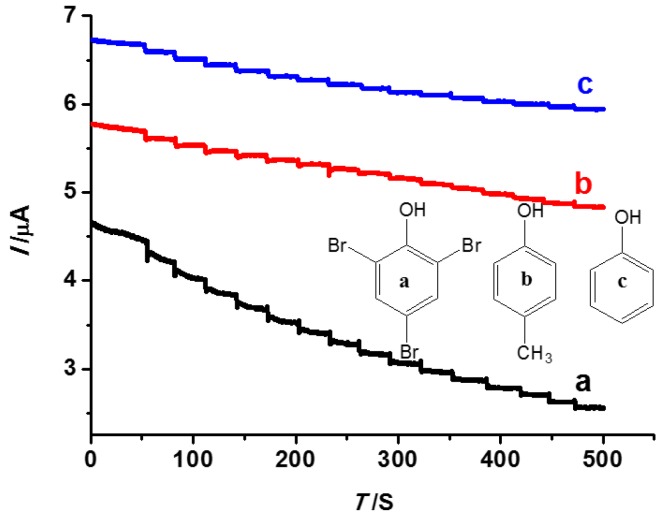
AC of the INS to different substances (**a**: TBP; **b**: 4-methyl-phenol; and **c**: phenol).

In order to investigate the selectivity of INS, besides TBP, some species, such as phenol and 4-methyl-phenol, were employed as interference for selectivity tests by AC with the same operation as the test of the detection limit. As shown in Figure 10, with the addition of different substances, the sensor for TBP is different from the other two interferences. The result showed that the recognition sites of TBP were formed in the INS, which played an important role in the process of recognition. The selective recognition is based on the interaction between the template and the imprinting sites. The recognition sites formed in the polymerized film have the capability to distinguish target molecules through their size, shape, and functional group distribution [42]. Moreover, the current value of TBP is always lower than phenol or 4-methyl-phenol. TBP bound to the polymer film, occupied the binding sites, and blocked the reduction of the ferricyanide species; therefore, much lower currents have been observed as in the case of the K_3_[Fe(CN)_6_] solution.

### 3.6. Regeneration and Stability

**Figure 11:**
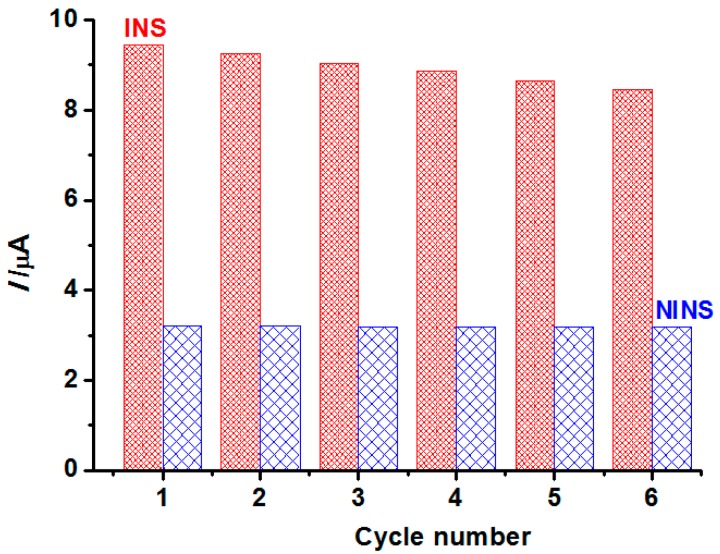
The regeneration of the INS/NINS.

Regeneration is one of the most important properties for the application of the imprinted sensor. Therefore, the sensor was immersed in ethanol by shaking for 3 h, and back to the initial state. DPV was applied in the solutions containing 5 mmol·L^−1^ K_3_[Fe(CN)_6_] and 5.0 × 10^−6^ mol·L^−1^ TBP. The cycle was repeated using the above description. As shown in Figure 11, the current value of INS is much more than NINS, although the NINS show almost no change. The current value of INS changed, but not much for six trials, which indicates good regeneration performance. A small current change of INS is possible that some recognition cavities might be blocked after regeneration or can be destroyed after rewashing and, thus, they no longer matched the template molecule.

As in [41], when not in use, the sensor can be simply protected in the electrode plastic cap filled with nitrogen, and stored at 4 °C in a refrigerator. The INS retained about 90% of its initial effect after 45 days of storage. The modified electrode exhibited good stability.

## 4. Conclusions

To determine TBP, a new approach for the fabrication of the TBP-MIP sensor was presented by combining a molecular imprinting technique and electrospinning. The modified electrode exhibited a low detection limit (0.21 µg·L^−1^), good recognition, and good regeneration performance. Compared with chromatography, LOD obtained from the experiment is outperformed more than GC (0.5 µg·L^−1^). In addition, the fabrication procedure is simple, rapid, and inexpensive. We believe that our strategy may be instructive to the determination of other phenolic pollutants, which are insoluble in water.

## References

[1] Birnbaum L.S., Staskal D.F. (2004). Brominated flame retardants: Cause for concern?. Environ. Health Perspect..

[2] Alaee M., Arias P., Sjödin A., Bergman Å. (2003). An overview of commercially used brominated flame retardants, their applications, their use patterns in different countries/regions and possible modes of release. Environ. Int..

[3] De Wit C.A. (2002). An overview of brominated flame retardants in the environment. Chemosphere.

[4] Thomsen C., Lundanes E., Becher G. (2002). Brominated flame retardants in archived serum samples from Norway: A study on temporal trends and the role of age. Environ. Sci. Technol..

[5] Covaci A., Harrad S., Abdallah M.A.E., Ali N., Law J., Herzke D., de Wit C.A. (2002). Novel brominated flame retardants: A review of their analysis, environmental fate and behaviour. Environ. Int..

[6] De Wit C.A., Herzke D., Vorkam K. (2010). Brominated flame retardants in the arctic environment—Trends and new candidates. Sci. Total Environ..

[7] Eriksson J., Jakobsson E. (1998). Decomposition of tetrabromobisphenol A in the presence of UV-light and hydroxyl radicals. Organohalogen Compd..

[8] Vetter W., Janussen D. (2005). Halogenated natural products in five species of Antarctic sponges: Compounds with POP-like properties?. Environ. Sci. Technol..

[9] Nichkova M., Marco M.P. (2006). Biomonitoring human exposure to organohalogenated substances by measuring urinary chlorophenols using a high-throughput screening (HTS) immunochemical method. Environ. Sci. Technol..

[10] Nichkova M., Germani M., Marco M.P. (2008). Immunochemical analysis of 2,4,6-tribromophenol for assessment of wood contamination. J. Agric. Food Chem..

[11] Cantón R.F., Sanderson J.T., Letcher R.J., Bergman A., van den Berg M. (2005). Inhibition and induction of aromatase (CYP19) activity by brominated flame. Toxicol. Sci..

[12] Deng J., Liu C.S., Yu L.Q., Zhou B.S. (2010). Chronic exposure to environmental levels of tribromophenol impairs zebrafish reproduction. Toxicol. Appl. Pharm..

[13] Blythe J.W., Heitz A., Joll C.A., Kagi R.I. (2006). Determination of trace concentrations of bromophenols in water using purge-and-trap after *in situ* acetylation. J. Chromatogr. A.

[14] Polo M., Llompart M., Garcia-Jares C., Gomez-Noya G., Bollain M.H., Cela R. (2006). Development of a solid-phase microextraction method for the analysis of phenolic flame retardants in water samples. J. Chromatogr. A.

[15] Huang J.T., Alquie L., Kaisa J.P., Reed G., Gilmor T., Vas G. (2012). Method development and validation for the determination of 2,4,6-tribromoanisole, 2,4,6-tribromophenol, 2,4,6-trichloroanisole, and 2,4,6-trichlorophenol in various drug products using stir bar sorptive extraction and gas chromatography–tandem mass spectrometry detection. J. Chromatogr. A.

[16] Gong K.P., Zhu X.Z., Zhao R., Xiong S.X., Mao L.Q., Chen C.F. (2005). Rational attachment of synthetic triptycene orthoquinone onto carbon nanotubes for electrocatalysis and sensitive detection of thiols. Anal. Chem..

[17] Agarwal S., Greiner A., Wendorff J.H. (2013). Functional materials by electrospinning of polymers. Prog. Polym. Sci..

[18] Moayeri A., Ajji A. (2015). Fabrication of polyaniline/poly(ethylene oxide)/non- covalently functionalized graphene nanofibers via electrospinning. Synth. Met..

[19] Ahmed F.E., Lalia B.S., Hashaikeh R. (2015). A review on electrospinning for membrane fabrication: Challenges and applications. Desalination.

[20] Zheng B.Z., Liu G.Y., Yao A.W., Xiao Y.L., Du J., Guo Y., Xiao D., Hu Q.M., Choi M.F. (2014). A sensitive AgNPs/CuO nanofibers non-enzymatic glucose sensor based on electrospinning technology. Sens. Actuators B Chem..

[21] Ji Y.T., Yan C.C., Yu H., Chen L.Z., Dong F.C. (2014). One-step fabrication of ammonia sensor by electrospinning PS-*b*-PMA nanofibers on quartz crystal microbalance. Sens. Actuators B Chem..

[22] Osmani Q., Hughes H., McLoughlin P. (2012). Probing the recognition of molecularly imprinted polymer beads. J. Mater. Sci..

[23] Chen L., Xu S., Li J. (2011). Recent advances in molecular imprinting technology: Current status, challenges and highlighted applications. Chem. Soc. Rev..

[24] Tiwari M.P., Prasad A. (2015). Molecularly imprinted polymer based enantioselective sensing devices: A review. Anal. Chim. Acta.

[25] Augusto F., Hantao L.W., Mogollon N., Braga S. (2013). New materials and trends in sorbents for solid-phase extraction. TrAC Trends Anal. Chem..

[26] Nicolescu V., Meouche W., Branger C., Margaillan A., Sarbu A., Fruth V., Donescu D. (2013). A new microemulsion approach for producing molecularly imprinted polymers with selective recognition cavities for gallic acid. Polym. Int..

[27] Ma Y., Pan G.Q., Zhang Y., Guo X.Z., Zhang H.Q. (2013). Narrowly dispersed hydrophilic molecularly imprinted polymer nanoparticles for efficient molecular recognition in real aqueous samples including river water, milk, and bovine serum. Angew. Chem. Int. Ed..

[28] Huynh T.P., Chandra B.K.C., Sosnowska M., Sobczak J.W., Nesterov V.N., D’Souza F., Kutner W. (2015). Nicotine molecularly imprinted polymer: Synergy of coordination and hydrogen bonding. Biosens. Bioelectron..

[29] Gupta V.K., Yola M.L., Atar N. (2014). A novel molecular imprinted nanosensor based quartz crystal microbalance for determination of kaempferol. Sens. Actuators B Chem..

[30] Tadi K.K., Motghare R.V., Ganesh V. (2014). Electrochemical detection of sulfanilamide using pencil graphite electrode based on molecular imprinting technology. Electroanalysis.

[31] Anirudhan T.S., Alexander S. (2015). Design and fabrication of molecularly imprinted polymer-based potentiometric sensor from the surface modified multiwalled carbon nanotube for the determination of lindane (γ-hexachlorocyclohexane), an organochlorine pesticide. Biosens. Bioelectron..

[32] Luo J., Cong J., Liu J., Gao Y., Liu X. (2015). A facile approach for synthesizing molecularly imprinted graphene for ultrasensitive and selective electrochemical detecting 4-nitrophenol. Anal. Chim. Acta.

[33] Ma X., Chen Z., Chen X., Chen R., Zheng X. (2011). Preparation of imprinted PVB/β-CD nanofiber by electrospinning technique and its selective binding abilities for naringin. Chin. J. Chem..

[34] Ma X., Liu J., Zhang Z., Wang L., Chen Z., Xiang S. (2013). The cooperative utilization of imprinting, electro-spinning and a pore-forming agent to synthesize β-cyclodextrin polymers with enhanced recognition of naringin. RSC Adv..

[35] Ma X., Wu D., Huang L., Wu Z., Xiang S., Chen S. (2015). Sensing 2,4,6-tribromophenol based on molecularly imprinted technology. Monatshefte Chem..

[36] Ma X., Liu J., Wu D., Wang L., Zhang Z., Xiang S. (2016). Ultrasensitive sensing tris (2,3-dibromopropyl) isocyanurate based on the synergistic effect of amino and hydroxyl groups of molecularly imprinted poly (*o*-aminophenol) film. New J. Chem..

[37] Chronakis I.S., Milosevic B., Frenot A., Ye L. (2006). Generation of molecular recognition sites in electrospun polymer nanofibers via molecular imprinting. Macromolecules.

[38] Chronakis I.S., Alexandra J., Bengt H., Ye L. (2006). Encapsulation and selective recognition of molecularly imprinted theophylline and 17 β-estradiol nanoparticles within electrospun polymer nanofibers. Langmuir.

[39] Liu F., Liu Q., Zhang Y., Liu Y., Wan Y., Gao K., Huang Y., Xia W., Wang H., Shi Y. (2015). Molecularly imprinted nanofiber membranes enhanced biodegradation of trace bisphenol A by pseudomonas aeruginosa. Chem. Eng. J..

[40] Zhai Y., Wang D., Liu H., Zeng Y., Yin Z. (2015). Electrochemical molecular imprinted sensors based on electrospun nanofiber and determination of ascorbic acid. Anal. Sci..

[41] Li S., Du D., Huang J., Tu H., Yang Y., Zhang A. (2013). One-step electrodeposition of a molecularly imprinting chitosan/phenyltrimethoxysilane/AuNPs hybrid film and its application in the selective determination of p-nitrophenol. Analyst.

[42] Liu Y.T., Deng J., Xiao X.L., Ding L., Yuan Y.L., Li H., Li X.T., Yan X.N., Wang L.L. (2011). Electrochemical sensor based on a poly(para-aminobenzoic acid) film modified glassy carbon electrode for the determination of melamine in milk. Electrochim. Acta.

